# Tracheostomy Practice in the Italian Intensive Care Units: A Point-Prevalence Survey

**DOI:** 10.3390/medicina61010087

**Published:** 2025-01-07

**Authors:** Raffaele Merola, Maria Vargas, Filippo Sanfilippo, Marco Vergano, Giovanni Mistraletti, Luigi Vetrugno, Gennaro De Pascale, Elena Giovanna Bignami, Giuseppe Servillo, Denise Battaglini

**Affiliations:** 1 Anesthesia and Intensive Care Medicine, Department of Neurosciences, Reproductive and Odontostomatological Sciences, University of Naples Federico II, 80138 Naples, Italy; vargas.maria82@gmail.com (M.V.); maria.vargas@unina.it (G.S.); 2 Department of General Surgery and Medico-Surgical Specialties, School of Anaesthesia and Intensive Care, University of Catania, 95124 Catania, Italy; filipposanfi@yahoo.it; 3 Department of Anesthesia and Intensive Care, San Giovanni Bosco Hospital, 10154 Torino, Italy; marco.vergano@aslcittaditorino.it; 4 SC Rianimazione e Anestesia, Ospedale Civile di Legnano, Azienda Socio Sanitaria Territoriale (ASST) Ovest Milanese, 20025 Milan, Italy; giovanni.mistraletti@unimi.it; 5 Department of Anesthesiology, Critical Care Medicine and Emergency, Department of Medical, Oral and Biotechnological Sciences, “G. d’Annunzio” University Chieti-Pescara, 66013 Chieti, Italy; luigi.vetrugno@unich.it; 6 Dipartimento di Scienze Biotecnologiche di Base, Cliniche Intensivologiche e Perioperatorie, Università Cattolica del Sacro Cuore, 20123 Rome, Italy; gennaro.depascalemd@gmail.com; 7 Dipartimento di Scienze Dell’Emergenza, Anestesiologiche e Della Rianimazione, Fondazione Policlinico Universitario A. Gemelli IRCCS, 00168 Rome, Italy; 8 Anesthesiology, Critical Care and Pain Medicine Division, Department of Medicine and Surgery, University of Parma, 43121 Parma, Italy; elenagiovanna.bignami@unipr.it; 9 Department of Surgical Sciences and Integrated Diagnostics (DISC), University of Genoa, 16126 Genova, Italy; battaglini.denise@gmail.com; 10 Anesthesia and Intensive Care, IRCCS Ospedale Policlinico San Martino, 16132 Genova, Italy

**Keywords:** intensive care unit (ICU), mechanical ventilation, outcomes, point prevalence, tracheostomy

## Abstract

*Background and Objectives*: A tracheostomy is a frequently performed surgical intervention in intensive care units (ICUs) for patients requiring prolonged mechanical ventilation. This procedure can offer significant benefits, including reduced sedation requirements, improved patient comfort, and enhanced airway management. However, it is also associated with various risks, and the absence of standardized clinical guidelines complicates its implementation. This study aimed to determine the prevalence of tracheostomy among ICU patients, while also evaluating patient characteristics, complication rates, and overall outcomes related to the procedure. *Materials and Methods*: We conducted an observational, cross-sectional, point-prevalence survey across eight ICUs in Italy. Data were collected over two 24 h periods in March and April 2024, with a focus on ICU characteristics, patient demographics, the details of tracheostomy procedures, and associated complications. *Results*: Among the 92 patients surveyed in the ICUs, 31 (33.7%) had undergone tracheostomy. The overall prevalence of tracheostomy was found to be 9.1%, translating to a rate of 1.8 per 1000 admission days. The mean age of patients with a tracheostomy was 59.5 years (SD = 13.8), with a notable predominance of male patients (67.7%). Neurological conditions were identified as the most common reason for ICU admission, accounting for 48.4% of cases. Tracheostomy procedures were typically performed after a mean duration of 12.9 days of mechanical ventilation, primarily due to difficulties related to prolonged weaning (64.5%). Both early and late complications were observed, and 19.35% of tracheostomized patients did not survive beyond one month following the procedure. The average length of stay in the ICU for these patients was significantly extended, averaging 43.0 days (SD = 34.3). *Conclusions*: These findings highlight the critical role of tracheostomy in the management of critically ill patients within Italian ICUs. The high prevalence and notable complication rates emphasize the urgent need for standardized clinical protocols aimed at optimizing patient outcomes and minimizing adverse events. Further research is essential to refine current practices and develop comprehensive guidelines for the management of tracheostomy in critically ill patients.

## 1. Introduction

A tracheostomy is a procedure routinely performed in intensive care units (ICUs) to facilitate airway management in patients who are experiencing respiratory failure and require prolonged mechanical ventilation support [1]. Tracheostomy appears to reduce the risk of death, the occurrence of ventilator-associated pneumonia (VAP), and the duration of mechanical ventilation itself, ultimately leading to a decreased length of stay for patients in the intensive care setting [2,3,4,5,6]. Furthermore, tracheostomy reduces the necessity for sedative medications, increases patient comfort, promotes better oral hygiene, facilitates improved communication skills, and supports the ability of patients to swallow and consume food and fluids orally [4,7]. In addition to these benefits, tracheostomy serves as an alternative airway option for patients facing obstructions, particularly those arising from conditions like oral or upper airway cancers [8,9,10,11]. Finally, tracheostomy is known to enhance respiratory function by minimizing airway resistance, which optimizes the work of breathing for patients [12,13]. However, it is essential to acknowledge that tracheostomy is not without its risks and potential complications. These may include bleeding, infection at the wound site, tracheal stenosis, tracheomalacia, tracheoesophageal fistula, trachea–innominate artery fistula, accidental displacement of the tube, and, in rare cases, death [14,15]. Despite the above benefits, no clinical guidelines have been developed to outline best practice for this procedure [16]. Consequently, to date, the decision to proceed with a tracheostomy is typically grounded in a thorough clinical and prognostic assessment of individual patients, weighing the specific benefits against the potential risks involved in each case [17,18].

As the practice of tracheostomy continues to evolve, it is increasingly important to emphasize the necessity of generating comprehensive data regarding its use. Notably, the existing literature indicates that no point-prevalence surveys have been conducted to evaluate tracheostomized patients within the ICU setting thus far. Our study aims to address this gap by being the first point-prevalence survey focused on tracheostomy practices in ICU. The primary objective of our investigation is to determine the current prevalence of tracheostomy among patients admitted to the ICU. Additionally, we have secondary aims that include assessing the characteristics, complications, and outcomes associated with tracheostomized patients in this critical care environment.

## 2. Methods

### 2.1. Study Design

This was an observational, cross-sectional, 24 h point-prevalence study divided into two parts conducted in eight intensive care units in Italy. Part I of the survey was released on 4 March 2024 (basic module), and Part II of the survey was released on 4 April 2024 (follow-up module). The survey was created in the web platform Google Moduli, and the link to access the survey was disseminated via e-mail to each participant. Only one respondent from each ICU was allowed.

The study protocol was approved by the ethics committee of the University of Naples Federico II (no 134/2023, Approval Date: 27 April 2023).

### 2.2. Participants

A contact person was identified for each intensive care unit invited to participate. We included in the study the physicians interested in participating who had responded to the survey at the times and in the ways previously established (from 8 a.m. to 8 p.m. on 4 March and 3 April 2024, respectively).

### 2.3. Data Collection

Data on hospital characteristics (name of the hospital, country, type of hospital); ICU characteristics and practice (type of ICU, number of ICU beds, number of admitted patients/year in ICU, number of tracheostomies performed in 2022); and point-prevalence data (number of currently admitted patients in ICU, number of patients intubated, number of patients tracheostomized, number of patients intubated and mechanically ventilated, number of patients without oxygen support, number of patients with low-flow oxygen support, number of patients with high-flow oxygen support, number of patients with non-invasive ventilation, age and sex of each patient with a tracheostomy, diagnosis at admission of each patient with a tracheostomy, technique used for each tracheostomy, days from initiation of mechanical ventilation to tracheostomy, indication for tracheostomy, number of weaning attempts before performing a tracheostomy, type of tracheostomy tubes used for each patient, early complications during the tracheostomy procedure, general complications of tracheostomy) were collected at day one. Follow-up data on point-prevalence tracheostomy were collected one month after the first assessment.

### 2.4. Definitions

Number of ICU admissions: Number of ICU admissions in a given year (data from previous year if available) were considered. Admission rate was estimated, considering the average value.

Number of patient-days: Number of ICU patient-days in a given year (data from previous year if available).

Airway management: Tracheostomy, endotracheal intubation, and natural airway management were considered as airway management.

Ventilatory management: Invasive mechanical ventilation, non-invasive mechanical ventilation, high-flow oxygen therapy, low-flow oxygen support (e.g., Venturi mask, nasal cannulas, etc.), and no oxygen support were considered as ventilatory management.

Prolonged mechanical ventilation is defined as a period of 21 days or more.

Difficult/prolonged weaning is defined as weaning requiring ≥3 spontaneous breathing trials, failure of ≥3 weaning attempts, or duration of >7 days after the first attempt.

Early complications are defined as complications that occur during the performance of the tracheostomy.

Late complications are defined as complications that occur once the tracheostomy has been performed.

Stoma infections/inflammations have been defined as sign of inflammation and purulent discharge of the stoma.

### 2.5. Statistical Analysis

SPSS Statistics 20 was used for statistical analysis. Data are reported as number (n), percentage (%), mean (standard deviation = SD), or median (1st–3rd quartiles = IQR), as appropriate. We calculated the estimated average admission rate of patients per year, the prevalence of tracheostomies, and the rate of tracheostomies per admission day.

## 3. Results

### 3.1. Characteristics of Participating ICUs

Eight Italian ICUs participated in the point-prevalence survey. Table 1 shows the characteristics of the included ICUs. Four of the included ICUs have an approximate number of admitted patients/year of 301–600, three ICUs have 601–999, and one ICU has ≥1000. As of March 2024, the maximum total number of available ICU beds was 123, of which 75.6% were occupied at the time of first assessment. The number of tracheostomies performed in 2022 was 475. Considering an estimated average admission rate of 5202 patients/year, the prevalence of tracheostomies/year was 9.1%, with a rate of 1.8 tracheostomies per 1000 admission days.

### 3.2. Characteristics of Tracheostomized Patients

**Figure 1:**
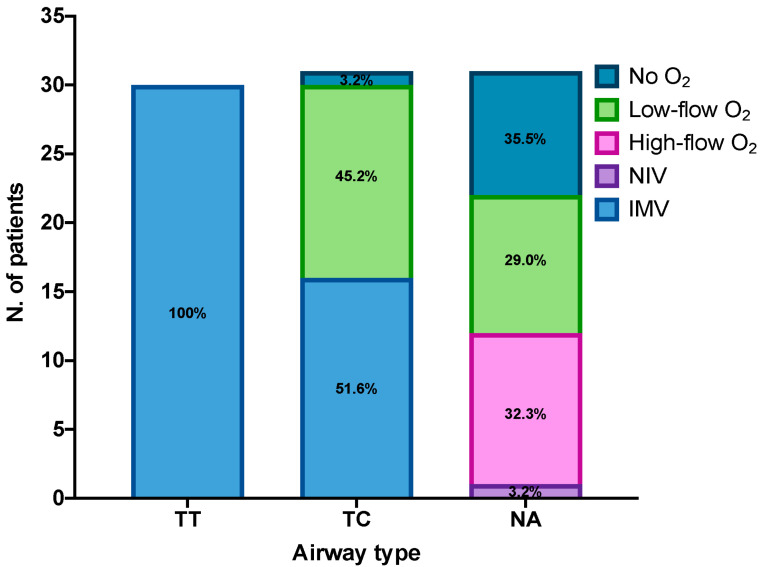
Characteristics of respiratory support according to type of airway. NIV = non-invasive ventilation, IMV = invasive mechanical ventilation, O_2_ = oxygen, TT = tracheal tube, TC = tracheostomy cannula, NA = natural airway.

At the time of first assessment, 92 patients were hospitalized in the ICUs, of whom 31 (33.7%) were breathing with a tracheostomy, 30 (32.6%) with a tracheal tube, and 31 (33.7%) with a natural airway. The characteristics of ventilation according to the type of airway are presented in Figure 1.

The mean age of tracheostomized patients was 59.5 (SD = 13.8) years, and 21 (67.7%) were males. The main reason for the ICU admission of tracheostomized patients was neurologic in 15 (48.4%), followed by surgical in 5 (16.1%), respiratory in 4 (12.9%), cardiovascular in 4 (12.9%), and for other reasons in 3 (9.7%) patients, of which two were admitted for polytrauma without neurological involvement. Tracheostomies were performed at a mean of 12.9 (SD = 13.8) days from the initiation of invasive mechanical ventilation. The mean number of weaning attempts before tracheostomy was 1.6 (SD = 1.5) times.

**Figure 2:**
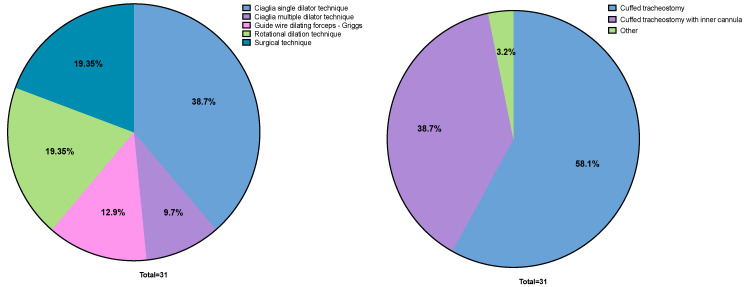
Type of tracheostomy technique and cannula used.

The main reason for tracheostomy was difficult/prolonged weaning in 20 (64.5%) patients, followed by inability to protect the airway in 7 patient (22.6%), and inability to cough and swallow in 4 (12.9%) patients. The most common technique utilized to perform tracheostomy was the Ciaglia single dilator, used in 12 (38.7%) patients, and the cannula most commonly used was cuffed, in 18 (58.1%) patients. The prevalence of the type of tracheostomy technique and cannula are reported in Figure 2.

**Figure 3:**
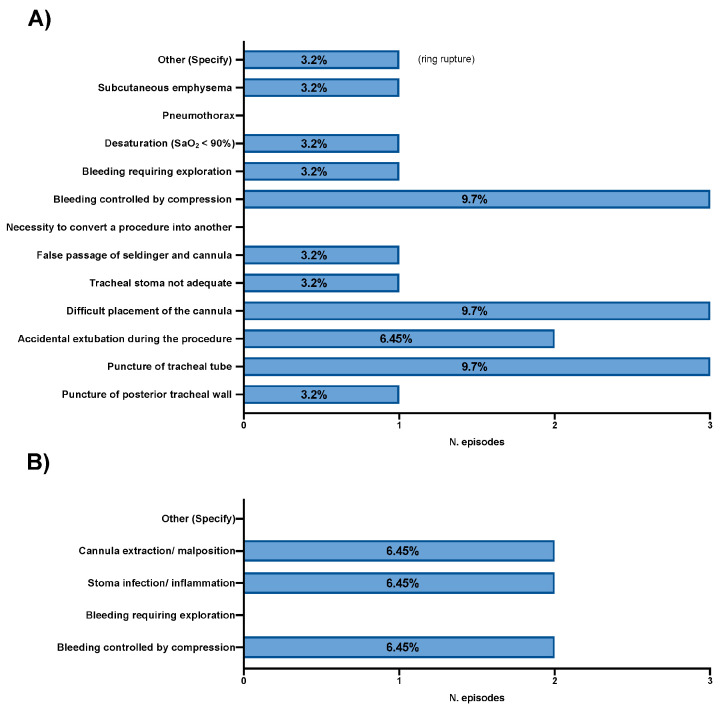
(**A**) Early complications of tracheostomies performed. (**B**) Late complications of tracheostomies performed. SaO_2_ = saturation of oxygen.

The early and late complications of tracheostomy are reported in Figure 3.

After one month, 6 (19.35%) tracheostomized patients did not survive. Of the patients alive, 8 (25.8%) went to a medical ward, 6 (19.35%) went to a rehabilitation and physical therapy ward, 3 (9.7%) went to a surgical ward, 1 (3.2%) was transferred to another ICU, while 7 (22.6%) patients were still in the ICU. The mean ICU LOS from admission to discharge or death was 43.0 (SD = 34.3, with two data points missing). All the patients who were discharged still had a tracheostomy, but only two were still invasively mechanically ventilated (the patient transferred to another ICU and the patient transferred to a rehabilitation ward).

## 4. Discussion

This study represents the first point-prevalence survey specifically focused on tracheostomy practices within ICUs across Italy. Our findings provide significant insights into various aspects of tracheostomized patients in these critical care environments, particularly regarding their prevalence, characteristics, complications, and overall outcomes. Notably, we found a tracheostomy prevalence of 9.1% among patients admitted to ICUs, along with a rate of 1.8 tracheostomies per 1000 admission days. These results emphasize the importance and relevance of tracheostomy as a critical procedure for managing patients who are critically ill and require prolonged mechanical ventilation support. The prevalence of tracheostomy and the rate of tracheostomy per hospital day observed in our study are consistent with the previous literature and help highlight the crucial role of tracheostomy in the management of critically ill patients undergoing prolonged mechanical ventilation [19,20,21].

The demographic profile of the tracheostomized patients in our study reveals a predominantly male population with a mean age of 59.5 years. Furthermore, it was found that neurological conditions were the most common reason for ICU admission among these patients. This finding highlights a wider trend seen in ICU settings, where patients with neurological issues frequently necessitate sophisticated airway management techniques, such as tracheostomy, to ensure proper respiratory support and care [22,23,24,25]. The mean time from the initiation of invasive mechanical ventilation to tracheostomy was 12.9 days. A notable percentage of these patients, specifically 64.5%, ultimately required a tracheostomy because they faced challenges related to difficult or prolonged weaning. These data emphasize the critical importance of determining the optimal timing for tracheostomy in the management of critically ill patients requiring prolonged mechanical ventilation [4,26,27,28]. However, the average number of attempts made to wean patients from mechanical ventilation prior to the placement of a tracheostomy indicates that implementing more systematic approaches for evaluating patients’ readiness for extubation could be beneficial. This suggests that refining our assessment methods may lead to better outcomes in the weaning process. Additionally, the frequent use of the Ciaglia single dilator technique along with cuffed tracheostomy tubes illustrates current practices that focus on enhancing both patient safety and the efficiency of the procedure. These modern techniques are designed to minimize complications and streamline the process, ultimately contributing to improved care for patients undergoing tracheostomy [29,30,31,32,33].

Despite significant advancements in the technique and practice of tracheostomy, complications continue to be a prominent concern, both early in and later after the procedure. Our findings mirror those of recent studies that report a troubling rate of complications, ranging from airway obstruction and infection to more complex issues such as tracheal stenosis and long-term respiratory difficulties [34]. This highlights the necessity for continuous monitoring and the implementation of effective management strategies aimed at reducing the incidence of such complications. Ongoing vigilance and proactive interventions are essential to ensure the safety and well-being of patients who undergo tracheostomy, ultimately improving their overall outcomes [35,36].

The survival rate of patients who underwent tracheostomy one month after the procedure reflects both the challenges and successes associated with this intervention. While it is noteworthy that 19.35% of patients did not survive, those who did experienced varied outcomes. A significant number of these patients were subsequently transferred to medical or rehabilitation departments, indicating a level of recovery and progress following the procedure. These findings present a novel perspective when compared to previous studies, which typically report higher mortality rates at follow-up. This suggests potential improvements in patient care and management strategies over time, highlighting the evolving landscape of tracheostomy outcomes [13]. Furthermore, the mean length of stay in the ICU was 43.0 days (SD = 34.3). This extended duration in the ICU underscores the complexity and severity of the patients’ medical conditions. Notably, all patients who were discharged required ongoing airway management, highlighting the chronic nature of their health issues. This situation emphasizes the importance of meticulous planning for post-ICU care strategies to ensure that patients receive appropriate support and monitoring as they transition out of the intensive care environment. Careful consideration of their ongoing needs is crucial for facilitating recovery and maintaining patient safety.

The findings of this study carry significant implications for clinical practice. Firstly, the prevalence of tracheostomy and the established outcomes indicate that it is an essential intervention within the ICU setting. However, these findings also emphasize the necessity for individualized management plans tailored to each patient’s unique clinical condition. Additionally, the complications observed in this study highlight the critical need for ongoing education and training for healthcare professionals who are involved in performing and managing tracheostomy procedures. Ensuring that medical staff are well trained and knowledgeable can help mitigate risks and improve patient outcomes, ultimately enhancing the quality of care provided to critically ill patients.

While our study offers important insights, it is important to acknowledge its limitations. First, the observational design and reliance on self-reported data from participating ICUs may introduce potential bias into our findings. Second, the brief time frame of the point-prevalence survey may not adequately capture the evolution of patients’ clinical statuses, which limits our ability to draw long-term conclusions regarding patient outcomes and complications. Third, prevalence surveys focus on existing cases and do not measure incidence, meaning that the dynamics of new tracheostomy placements or removals occurring during the survey period are not accounted for. Fourth, tracheostomy-related outcomes, such as complications and length of stay, may not be fully captured, especially for patients who had resolved issues prior to the survey date. Fifth, without longer-term follow-up data, it can be challenging to evaluate the efficacy of tracheostomies or the emergence of complications over time, which is crucial for understanding patient trajectories and outcomes. Finally, local variations in tracheostomy practices might affect both the prevalence and management of tracheostomy patients, potentially limiting the generalizability of our findings to other healthcare settings.

Future studies should prioritize longitudinal assessments of tracheostomized patients, examining not only the immediate outcomes associated with the procedure but also the long-term effects on quality of life and functional status. This approach will provide a more comprehensive understanding of the impact of tracheostomy over time. Additionally, conducting similar surveys across diverse geographic and clinical settings could enhance the generalizability of our findings. Such efforts would facilitate the development of robust, evidence-based guidelines for the management of tracheostomy, ultimately improving care for patients in various healthcare environments.

## 5. Conclusions

In summary, this point-prevalence survey underscores the critical role of tracheostomy in managing critically ill patients within Italian ICUs. While the advantages of tracheostomy are well established, our findings highlight an urgent need for standardized guidelines and protocols aimed at optimizing patient outcomes and reducing complications. Ongoing research in this field is essential for refining tracheostomy practices and enhancing the care provided to patients who require prolonged mechanical ventilation. By addressing these needs, we can improve the overall quality of care and support for this vulnerable patient population.

## Figures and Tables

**Table 1:** Characteristics of the included intensive care units.

ICU ID	Country	Type of Hospital	ICU Type	Maximum Number of ICU Beds	Approximate Number of Admitted Patients/Year	Number of Tracheostomies Performed in 2022
1	Italy	Academic	Mixed	20	301–600	42
2	Italy	Academic	Mixed	14	301–600	58
3	Italy	Academic	Surgical	8	301–600	22
4	Italy	Academic	Mixed	8	301–600	18
5	Italy	Tertiary	Surgical, Medical	13	601–999	68
6	Italy	Academic	Mixed, Surgical, Medical, Neurological	28	601–999	75
7	Italy	Academic	Medical	16	601–999	120
8	Italy	Academic	Surgical, Medical	16	≥1000	72

## Data Availability

The original contributions presented in this study are included in the article. Further inquiries can be directed to the corresponding author.
